# Prevalence of Helicobacter Pylori in Endoscopic Gastric Biopsies of Chronic Gastritis Patients at A Tertiary Care Centre

**DOI:** 10.31729/jnma.5210

**Published:** 2020-08-31

**Authors:** Archana Tiwari, Ramji Rai, Prahar Dahai, Sudeep Regmi

**Affiliations:** 1 Department of Pathology, Lumbini Medical College and Teaching Hospital, Tansen, Palpa, Nepal; 2 Department of Pathology, Manipal Medical College and Teaching Hospital, Pokhara, Nepal

**Keywords:** *endoscopy*, *gastritis*, *H. pylori*

## Abstract

**Introduction:**

Presence and severity of Helicobacter pylori *(H. pylori)* colonization is an important factor to decide the treatment of chronic gastritis. This study aimed to find the prevalence of H. pylori colonization in chronic gastritis patients.

**Methods:**

This descriptive,cross-sectional study was carried out at the tertiary care center in the the western region of Nepal among the dyspeptic patients undergoing endoscopic gastric biopsy from October 2018 to March 2020 after approval from the Institutional review committee. Convenience sampling was done to reach the sample size. Two hundred fifty cases were included in the study. Data were recorded in proforma and Data analysis was done in the statistical package for social sciences (SPSS 16.0). The severity of *H. pylori* colonization and gastritis was graded using the Updated Sydney System.

**Results:**

All cases showed chronic gastritis on histopathology. *H. pylori* were seen in 150 (60%) of cases. Mild, moderate, and severe *H. pylori* infection was seen in 59, 78, and 13 cases respectively. Out of 59 mild *H. pylori* cases, 35 (59.32%) had mild chronic inflammation; out of 78 moderate *H. pylori* cases 51 (65.38%) had moderate chronic inflammation and among 13 severe *H. pylori* cases, 10 (76.92%) had severe chronic inflammation.

**Conclusions:**

Prevalence of *H. pylori* colonization is high in chronic gastritis and there is a parallel increase in the severity of gastritis with an increase in the severity of *H. pylori* load.

## INTRODUCTION

Helicobacter pylori (H. pylori) is a bacterium, first discovered in 1982 by Robin Warren and Barry Marshall.^1^ Since its discovery, it has been associated with a wide spectrum of gastro-duodenal diseases including gastritis, gastro-duodenal ulcers, mucosa associated lymphoid lymphoma and gastric adenocarcinoma.^2^ H. pylori is the most common human infection of the stomach and it is also common in Nepal.^3,4^ The prevalence and histopathology of H. pylori induced gastritis has been studied globally.

Gastritis is a common health problem among the Nepalese population and endoscopy along with gastric biopsies are frequently performed at our institution. Data regarding the prevalence of H. pylori and histomorphology of chronic gastritis in relation to H. pylori infection is sparse in our country.

This study aimed to find the prevalence of H. pylori colonization in chronic gastritis patients.

## METHODS

This descriptive cross-sectional study was conducted at the Department of Pathology, Lumbini Medical College, and Teaching Hospital from October 2018 to March 2020 over eighteen months. This study was approved by the Institutional Review Committee (IRC NoIRC- LMC 12-H/018) of LMCTH, Tansen, Palpa,Nepal. Both the verbal and written informed consents were taken from each of the participants (or their guardians). The study population is patients who have undergone upper gastrointestinal (UGI) endoscopic evaluation. Patients with dyspeptic symptoms who underwent UGI endoscopy and gastric biopsy were included in the study. Biopsies of patients who were receiving or had received H. pylori eradication treatment within one month and neoplastic cases were not included in the study. Biopsies of patients of all ages and both sexes were included in the study. Age and sex of the patients, detailed clinical history, and upper gastrointestinal endoscopy findings were obtained from the requisition form.

Convenience sampling was done and the minimum sample size was calculated using the formula,

$$\begin{array}{ll} \text{n} & \text{= }{\text{Z}}^{\text{2}}\times \text{p }\times \text{q}/{\text{d}}^{\text{2}}\\ & \text{= }{(\text{1.96)}}^{\text{2}}\times \text{0.38}\times (1-0.38)/{(\text{0.1)}}^{\text{2}}\\ & \text{= }91 \end{array}$$

Where,

Z = 1.96 at 95% Cl.
p = prevalence of H. pylori infection (Miftahussurur et al 4), 38.4%
q = 1-p
d = margin of error, 10%

The minimum sample size was calculated to be 91.

Histopathological diagnosis was made based on antral biopsy sections findings, as an antral biopsy specimen was received in all the cases and considering the fact that in subjects with intact acid secretion, H. pylori, in particular, colonizes the gastric antrum. Biopsy specimens were fixed in 10 % formalin routinely processed and paraffin blocks were sectioned at 3-4 μm thickness. Two set sections of each specimen were made and both the sections were routinely stained with hematoxylin and eosin(H & E) and Giemsa stain respectively. Both sections from each specimen were evaluated under light microscopy by a single pathologist. The histomorphology of sections was studied on routine H & E stained sections. Giemsa stained sections were studied for the identification of H. pylori.

The presence of the morphological variables (H. pylori density; neutrophilic activity; mononuclear cell (MNC) infiltrations) was determined using the semi-quantitative method of scoring with a Visual Analogue Scale according to the updated Sydney System Classification of chronic gastritis,^5^ and scored 0-3 (0-none; 1-mild; 2-moderate and 3-marked) to each morphological variable. Intestinal metaplasia, glandular atrophy was not graded and was simply recorded as present or absent.

Selection bias and interpretation bias was minimized as possible. Data were recorded in the proforma form. The data was then coded and entry was done in the statistical package for the social sciences (SPSS) version 16.0. The data was processed and analyzed by using simple descriptive statistics; in terms of percentage and frequency.

## RESULTS

A total of 250 gastric biopsy specimens, that met the inclusion criteria were studied. On histological examination, H. pylori were detected in 150 (60%) patients and 100 (40%) of patients were H. pylori negative.

The average age of the total population was 43.43 years (standard deviation (SD) 15.13) with age ranging 12-81 years. One hundred sixty (64%) samples were obtained from males and 90(36%) samples were from females, with male to female ratio 1.77:1. The average age of male patients was 44.19 (SD 15.38) years, and the average age of women was 42.08 (SD 14.66) years. The mean age of patients in H. pylori positive group was 44.03 (SD 15.22) years and mean age in H. pylori negative group was 42.52 (SD 15.01) years. Male to female ratio in H. pylori positive and H. pylori negative group was 1.88:1 vs 1.63:1 respectively.

Chronic inflammation (Mononuclear cell infiltrations) was found in all of the cases of both H. pylori positive and negativecases. The severity ofchronic inflammation was seen high amongH. pylori positive cases. Moderate inflammation predominated in H. pylori positive groupand was found in 75 (50%) cases. In H. pylori negative group, mild inflammation predominated and was found in 51 (51 %) cases. In H. pylori positive samples, Neutrophilic activity was not seen in 14 (9.33%) samples, in the remaining 136 (90.66%) specimens, neutrophilic activity was present. In H. pylori negative samples, neutrophilic activity was not seen in 78 (78%) samples, whilet he remaining 22 (22%) cases showed neutrophilic activity. Distribution of histomorphological features gastritis among H. Pylori positive and negative groups is shown (Table 1).

**Table 1:** Showing the distribution of histomorphological features among H. Pylori positive and negative groups.

H. pylori status (n)	Chronic inflammation (MNC infiltration) n (%)			Neutrophilic activity present n (%)	Intestinal metaplasia n (%)	Glandular atrophy n (%)
	Mild	Moderate	Marked			
Positive (150)	54 (36%)	75 (50%)	21 (14%)	136 (90.66%)	7 (4.6%)	4 (2.6%)
Negative (100)	51 (51%)	46 (46%)	3 (3%)	22 (22%)	0 (0%)	0 (0%)

Out of 150 H. pylori positive cases, mild H. pylori colonization was seen in 59 (39.3%) samples, moderate colonization was seen in 78 (52%), and marked colonization was seen in 13 (8.7%) specimens. Of 59 mild H. pylori gastritis, mildchronic inflammation predominated and was found in 35 (59.32%) cases. Among 78 moderate H. pylori positive group, moderate chronic inflammation predominated and was found in 51 (65.38%) cases. Of 13 marked grade of H. pylori positive group, marked inflammation predominated and was found in 10 (76.92%) cases. Distribution of different grades of chronic inflammation in different severity of H. pylori colonization is shown in (Table 2).

**Table 2:** Distribution of different grades of chronic inflammation in different severity of H. pylori colonization.

The density of H. pylori	Chronic inflammation (Mononuclear cell infiltrations) n (%)			
	mild	moderate	Marked	Total n (%)
Mild	35 (59.32%)	21 (35.59%)	3 (5.08%)	59 (100%)
Moderate	19 (24.35%)	51 (65.38%)	8 (10.25%)	78 (100%)
Marked	0	3 (23.07%)	10 (76.92%)	13 (100%)

Among 59 mild H. pylori positive group, mild neutrophilic activity predominated and was seen in 29 (49.15 %) cases. Out of 78 moderate H. pylori positive group, moderate neutrophilic activity predominated and was found in 39 (50%) cases. Out of 13 marked H. pylori positive group,marked neutrophilic activity predominated and was found in 9 (69.23%). Distribution of different grades of neutophilic activity in different severity of H. pylori colonization is shown (Table 3).

**Table 3:** Distribution of different grades of neutophilic activity in different severity of H. pylori colonization.

Density of H. pylori	Neutrophilic activity n (%)				Total n (%)
	no	Mild	Moderate	Marked	
Mild	10 (16.94%)	29 (49.15%)	19 (32.2%)	1 (1.69%)	59 (100%)
Moderate	4 (5.12%)	24 (30.76%)	39 (50%)	11 (14.1%)	78 (100%)
Marked	0 (0%)	1 (7.69%)	3 (23.07%)	9 (69.23%)	13 (100%)

## DISCUSSION

In this study, histopathological reporting was done in all cases on antral biopsies, as antrum was the most common site from which biopsies were received. For the consideration of the patient's comfort and operator's convenience, it was difficult to follow the extensive endoscopy biopsy protocol of five biopsy samples as per the updated Sydney system, from every patient. Eriksson et al have recommended that antrum is the most likely site of histopathological findings in gastritis.^6^ Similar to our study, Garg et al,^7^ Park et al,^8^ and Dhakhwa et al,^9^ have alsoconsidered their studies mainly on antral biopsies.

In our study, the age group affected by the varying grade of chronic Gastritis ranged from 12-81 years and the mean age was 43.43 years. This finding is similar to findings of other studies where the mean age was 47 years.^7,10^

A predominance of a male having chronic inflammation has been noticed in this study with an M: Fratio of 1. 77:1 which is similar to the study ofGarg et al, Park et al, Chen et al, and Pruthi et al, where they reported an M:F ratio of 2.1:1,2.8:1, 1.8: 1, 2.3:1 and respectively.^7,8,11,12^ H .pylori infection was more common in males than females, which is at par with the result of many previous studies.^7,8,12,13^ However, this finding is contrary to the study by Maharjan et al, where females were found to have a higher rate of H. pylori infection .^14^

In this study, H. pylori infection was identified in 150 (60%) cases out of 250 (100%) specimens included in the study. Similar figures are seen in the study conducted by Park et al.^8^ In previous studies, the prevalence of H. pylori varied from 43.66% of Garg et al to 93.7% of Hassan et al .^7,10^

This variation in the prevalence of H. pylori may be due variation in biopsy sampling sites for varying intragastric colonization, host immune response, level of acid production, and prior treatment with proton pump inhibitors or anti H. pylori antimicrobial agents. Multiple site sampling may be needed to improve results.

Chronic Gastritis (Mononuclear cell infiltration) was seen in 250 (100%) cases in the present study. Various other studies have shown findings similar to this study.^7,8,15^ Majority of inflammation in this study were of moderate grade (48.8%) followed by mild 42% and marked 9.6%, which is similar to the other studies.^8,14,15^ In contrary to this, a study by Garg et.al found that majority of inflammation was mild.^7^ Moreover H. pylori was detected in 150 (60%) cases of Chronic gastritis that is consistent with a previous study.^14^

In this study, we have seen the severity of chronic inflammation parallels the severity of H. pylori density. Mild chronic inflammation predominated in mild H. pylori group. Moderate chronic inflammation predominated inthe moderate H. pylori group. Severe chronic inflammation predominated in the severe H. pylori group. This is at par with the results of other studies,^7,15–19^, however, it is contrary with the study of park et., as they found no association between the grade of mononuclear cell infiltration and density of H. pylori.^8^

Overall, Neutrophilic activity was seen in 158 (63.2%) cases out of a total of 250 (100%) cases. This is lower than the studies by Park et al and A Hussein et al; where neutrophilic activity was seen in 78.75% and 84% cases respectively.^8,15^ However, the result in the present study outnumbered the observation of previous studies by Garg et al (33.33 % ), Hassan et al (31.84%), and Maharjan et al (33.6%).^7,10,14^

In H. pylori negative group (100 cases), neutrophilic activity was seen only in 22 (22%). In H. pylori positive group (150 cases), neutrophilic activity was seen only in 136 (90.66%) cases. In previous studies, the presence of neutrophilic activity in H. pylori positive cases was seen from low to as high as 100% cases. The neutrophilic activity was present in 100 % H .pylori positive cases in a study by A Hussein et al and Dhakhwa et.al.^9,15^ In the study conducted by Maharjan et al,^14^ Neutrophilic activity was seen in (40.7%) of H. pylori positive cases.

Similar to chronic inflammation, the severity of neutrophilic activity parallels the severity of H. pylori density. Mild neutrophilic activity predominated in a mild H. pylori group. Moderate neutrophilic activity predominated in moderate H. pylori group. Severe neutrophilic activity predominated in the severe H. pylori group. This finding is similar to previous other studies.^7,14–17^ However, Park et al,^8^ observed nostatistical association between neutrophilic activity and H. pylori concentration.

In H. pylori positive cases, glandular atrophy was seen in 4 (2.6%) and intestinal metaplasia was seen in 7 (4.6 %) cases in the presentstudy. No cases of glandular atrophy or intestinal metaplasia were seen in H. pylori negative cases. In contrary to this finding Dakhwa et al, Pruthi et al, and Maharjan et al found glandular atrophy and intestinal metaplasia in both H. pylori negative and positive cases.^9,12,14^

The limitation of this study is that only antral biopsy was considered for this study and the updated Sydney system was not strictly followed regarding sampling of gastric sites. Adherence to updated guidelinesof biopsy sites, considering the topographic distribution of H. Pylori, the diagnostic yield of gastric inflammatory conditions and H. pylori detection would have improved.

## CONCLUSIONS

This study concludes that the prevalence of H. pylori infection is high in chronic gastritis. There is a parallel increase in the severity of chronic inflammation (lymphoplasmacytic infiltration) and neutrophilic activity as the severity of H. pylori infection increases. Greater the density of H. pylori, the larger is the degrees of chronic inflammation and neutrophilic activity. Histomorphological search for H. pylori should be meticulously initiated if neutrophils are seen in the foveolar epithelium surface or glandular neck. Grades of H. pylori colonization can further guide chemotherapy regime, duration, and follow up to eradicate H. pylori and prevent complications related to it. Moreover, we also recommend to include multiple sites sampling protocol as far as possible to avoid false negative results.

## Conflict of Interest

**None.**
